# Influence of the Lignin Content on the Properties of Poly(Lactic Acid)/lignin-Containing Cellulose Nanofibrils Composite Films

**DOI:** 10.3390/polym10091013

**Published:** 2018-09-11

**Authors:** Xuan Wang, Yuan Jia, Zhen Liu, Jiaojiao Miao

**Affiliations:** 1 College of Chemical Engineering, Xi’an University, Xi’an 710065, China; jiayu_an_happy@126.com (Y.J.); liuzhen_lz@163.com (Z.L.); 2 School of Life Sciences, Northwestern Polytechnical University, Xi’an 710072, China; mjj.smile@163.com

**Keywords:** lignin-containing cellulose nanofibrils, poly(lactic acid) and composite films, lignin content, compatibility

## Abstract

Poly(lactic acid) (PLA)/lignin-containing cellulose nanofibrils (L-CNFs) composite films with different lignin contents were produced bythe solution casting method. The effect of the lignin content on the mechanical, thermal, and crystallinity properties, and PLA/LCNFs interfacial adhesion wereinvestigated by tensile tests, thermogravimetric analysis, differential scanning calorimetry (DSC), dynamic mechanical analysis, Fourier transform infrared spectroscopy (FTIR), and scanning electron microscopy (SEM). The tensile strength and modulus of the PLA/9-LCNFs (9 wt % lignin LCNFs) composites are 37% and 61% higher than those of pure PLA, respectively. The glass transition temperature (*T*_g_) decreases from 61.2 for pure PLA to 52.6 °C for the PLA/14-LCNFs (14 wt % lignin LCNFs) composite, and the composites have higher thermal stability below 380 °C than pure PLA. The DSC results indicate that the LCNFs, containing different lignin contents, act as a nucleating agent to increase the degree of crystallinity of PLA. The effect of the LCNFs lignin content on the PLA/LCNFs compatibility/adhesion was confirmed by the FTIR, SEM, and *T*_g_ results. Increasing the LCNFs lignin content increases the storage modulus of the PLA/LCNFs composites to a maximum for the PLA/9-LCNFs composite. This study shows that the lignin content has a considerable effect on the strength and flexibility of PLA/LCNFs composites.

## 1. Introduction

As a renewable bio-based polymer, poly(lactic acid) (PLA) is considered to be a promising alternative to petroleum-based plastics because of its excellent physical properties and thermal process ability [1,2]. However, its disadvantages are inherent brittleness, low degree of crystallization, and relatively poor thermal stability, which limits its wide application in packing, automotive, and biomedical fields [3,4]. Incorporating nanoparticle fillers as a reinforcing agent has been shown to improve the physical, thermal, and crystallization properties of PLA [5]. Cellulose nanocrystals (CNCs) have attracted academic and industrial interest as a reinforcing agent for PLA composites owing to their excellent strength, biodegradability, high specific surface area, and crystallization [6,7]. CNCs are mainly derived from the cellulose of lignin-free wood by acid hydrolysis. The high hydrophilicity of natural CNCs is because of the large amount of hydroxyl groups on their surface, which leads to poor interfacial interactions and compatibility between CNCs and hydrophobic PLA [8,9]. The tendency to form agglomerates upon incorporation into the PLA matrix prevents realization of the full potential of CNCs as a reinforcing phase [10].

Adding compatible materials is an effective approach to improve the dispersion of CNCs in the polymer matrix. Lignin improves the interfacial interaction in CNC/hydrophobic polymer composites because of its higher hydrophobicity than cellulose [11,12,13,14]. Furthermore, hydrogen bonds can be hindered by embedding lignin molecules between the cellulose chains, which results in less agglomeration compared with lignin-free CNCs [15]. Lignin is the second most abundant bio-based polymer on earth. It is composed of repeating phenyl propane units with aliphatic and aromatic hydroxyl groups and carboxylic acid groups, which plays a key role in improving the compatibility between CNCs and hydrophobic polymers through hydrogen bonding and van der Waals interactions [16,17,18,19]. The presence of lignin makes the dispersion and compatibility of CNCs better, and lignin-containing CNCs are expected to be an excellent reinforcing agent for PLA composites.

Actually, the lignin-containing CNFs have been studied in a lot of papers [20,21]. While, there are few papers recently published on lignin-containing CNFs based PLA biocomposites. Nair et al. [22] learned the improved mechanical, thermal, and barrier properties of polylactic acid bio-composites reinforced with nanocellulose fibrils with high lignin content; the different amount of high lignin nanocellulose fibrils (NCFHL) have made differences to the properties of PLA and the presence of lignin imparted a strong compatibility between the NCFHL and PLA matrix. Winter et al. [23] showed the reduced surface polarity, improved dispersion, and improved reinforcement efficiency of micro-fibrillated cellulose in poly(lactic acid), provided by residual lignin and hemicellulose content, compared to conventional microfibrillated cellulose (MFC) produced from bleached pulp.

In a recent study, we reported that lignin-containing cellulose nano-fibrils (LCNFs) can be dispersed in PLA by the solvent casting technique, and the thermal, mechanical, and crystallization properties of PLA are enhanced [24]. A lignin coating on the CNFs aids in initial dispersion and also prevents re-aggregation of cellulose nano-fibrils (CNFs) in the PLA matrix. The lignin on the CNF surface might make the PLA chains fold onto the LCNFs surface through improved interactions and compatibility, which also may allow for the efficient load transfer between the CNFs and the PLA matrix. The lignin content in the LCNFs is an important factor in studying the dispersion and compatibility improvement efficiency of LCNFs in PLA. In the present study, LCNFs with different lignin contents were dispersed in the PLA matrix by the solvent casting method, and their properties were investigated by Fourier transform infrared (FTIR) spectroscopy, thermogravimetric (TG) analysis, differential scanning calorimetry (DSC), dynamic mechanical analysis (DSC), and scanning electron microscopy (SEM). Compared with the researches of Nair and Winter, which focused on the performance of different amount of high lignin nano-cellulose fibrils and microfibrillated cellulose with residue lignin and hemicellulose in PLA matrix, respectively [22,23], our work aimed at the performance of PLA bio-composites with lignin-containing cellulose nano-fibrils from low lignin to high lignin content. Additionally, the effect of lignin content on the properties of PLA/LCNFs bio-composites has not been studied, so far. The current work demonstrates that the lignin content in the LCNFs plays an important role in improving the mechanical performance and process behavior of PLA composites.

## 2. Materials and Methods

### 2.1. Materials

L-PLA was provided by Shanghai Yisheng Industry, Ltd., Shanghai, China. The lignin-containing cellulose pulp boards (lignin contents of ~0, 6, 12, and 18 wt %) were purchased from a pulp and paper mill in Inner Mongolia, Orient Paper Plant Co., Ltd., Ulanchab, China. *N*,*N*-Dimethylacetamide (DMAc) and sulfuric acid (98 wt %) were obtained from Sinopharm Chemical Reagent Co., Ltd., Shanghai, China.

### 2.2. Preparation of the LCNFs Suspension 

The LCNFs were prepared by sulfuric acid hydrolysis and high-pressure homogenization of lignin-containing cellulose pulp boards, according to a previously reported method [24]. In a typical procedure, 5 g of pulp board was mixed with 100 mL of 15 wt % sulfuric acid at a constant speed for 4 h at 85 °C. The suspension was diluted with deionized water and then centrifuged at 4000 rpm for 10 min to concentrate the residue and to remove H^+^ and SO_4_^2−^ ions. The resultant precipitate was dispersed in DMAc by the solvent exchange method. The suspension was then further dispersed by homogeneous treatment (GEA Niro Soavi homogenizer, Parma, Italy, diameter of 10 mm, and process volume of 100 mL) at a pressure of 100 MPa for 10 cycles. A well-dispersed LCNFs suspension was then obtained. The LCNFs with different lignin contents were prepared by the same procedure, the lignin in the resulting LNCFs were tested and they are referred to as CNFs (0-LCNFs), 5-LCNFs, 9-LCNFs, and 14-LCNFs for lignin contents of ~0, 5, 9, and 14 wt %, respectively. The yield of the LCNFs from the pulp boards with acid hydrolysis and homogenization was about 60%.

### 2.3. Preparation of PLA/LCNFs Composite Films

The desired amount of 16 wt % DMAc solution of PLA was mixed with the LCNFs suspension by vigorous stirring with a magnetic stirrer (Shanghai, China). The mixture was sonicated and further stirred at 70 °C for 2 h. The suspension was then cast on glass with a scraper and dried at 80 °C for 30 min on an electric heating board. The obtained composite films were heated at 40 °C under a vacuum for 24 h to ensure that the solvent completely evaporated. By varying the added type of LCNFs, composite films with different lignin contents were prepared. The weight percentage of the LCNFs solid, in the PLA composite films, was 3 wt %.

### 2.4. Characterization 

The lignin content was measured by the half-scale kappa test method, which is based on the AS/NZS 1301.201.2002 method, Papro 1.106 kappa number, and TAPPI T236 standard [25]. The morphologies of the LCNFs samples were observed by TEM. A drop of a 0.01 wt % water suspension of LCNFs was deposited on a carbon-coated grid and negatively strained with 2% phosphotungstic acid. The images of the specimens were obtained with a Hitachi H-600 transmission electron microscope (Hitachi Limited, Tokyo, Japan) operated at an acceleration voltage of 80 kV. The initial contact angle (CA) measurements of the LCNFs were performed with three pure liquids with different polarities, namely, water, formamide, and ethylene glycol. The LCNFs–water suspensions were diluted to a 1 wt % solid content in DMAc and sonicated for 1 h at room temperature. Each suspension was poured into an over-pressurized filtration device equipped with a qualitative filter paper with particle retention of 12–15 μm to remove water and retain the fibrils. The CA was determined by a Harke-Space CA instrument (Beijing, China). The surface free energy of the LCNFs was determined by the calculation method provided in the Supporting Information. The FTIR spectra of the PLA/LCNFs composite films were obtained with a FTIR spectrometer (VERTES TOV, Bruker, Germany) over the wavenumber range 400–4000 cm^−1^. All of the samples were directly detected. The tensile properties of the composites were measured by a mechanical tester (ZB-WL300, Beijing, China) with a cross-head speed of 20 mm/min, a gauge length of 50 mm, and a 300 N load cell. Rectangular specimen strips 100 mm long, 15 mm wide, and 100 μm thick were tested. At least five measurements were performed for each sample and the data were averaged to obtain a mean value. TGA of the LCNFs and PLA/LCNFs composites was performed with a TG analyzer (TGA Q5000 IR, TA instrument, New Castle, DE, USA) under 100 mL/min nitrogen gas flow. Samples of about 6.0 mg were heated from room temperature (25 °C) to 500 °C at a heating rate of 10 °C/min. The cross-sections of the PLA/LCNFs composite films were observed with a Hitachi S-3400 scanning electron microscope (Tokyo, Japan) under an accelerating voltage of 15 kV. All of the samples were tensile broken in order to expose the internal structure (tensile specimens) before the examination, and the entire surface was sputtered with gold. The thermo mechanical properties of PLA and the PLA/LCNFs composites were determined with a DMA instrument (Q800, TA Instruments, New Castle, DE, USA). The tensile storage modulus and tan delta were determined at a frequency of 1 Hz, a strain rate of 0.05%, and a heating rate of 5 °C over the temperature range 0–100 °C. The test samples were prepared by cutting strips with a width of 10 mm and a length of 40 mm from the films. A TA Instruments Q2000 differential scanning calorimeter (New Castle, DE, USA) was used to record the DSC scans under N_2_. The sample (about 6–8 mg) was first heated from 20 to 200 °C at a heating rate of 10 °C/min and then kept at 200 °C for 5 min to remove the thermal history of the material. The sample was then cooled to 20 °C at 5 °C/min and kept at 20 °C for 5 min before heating to 200 °C again at the same heat rate. The degree of crystallinity *X_c_* was calculated with the following equation:

(1) $$X_{c}[\%]=[(\Delta H_{m}/\Phi_{PLA})/\Delta H_{m}^{0}]\times 100$$

where $\Delta H_{m}$ (J/g) is the enthalpy of fusion of the polymer composite, $\Delta H_{m}^{0}$ is the enthalpy of fusion of a PLA crystal of infinite size (assumed to be 93.6 J/g), and $\Phi_{PLA}$ is the fraction of PLA in the composite.

## 3. Results and Discussion

### 3.1. Properties of LCNFs with Different Lignin Content

**Figure 1:**
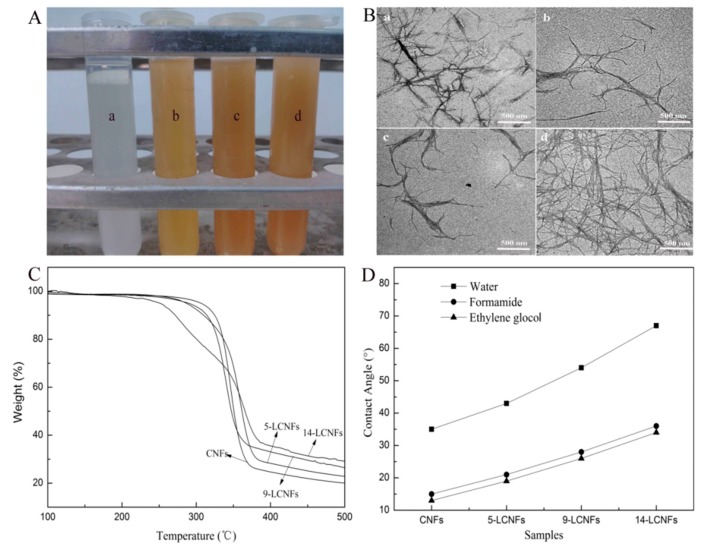
Properties of LCNFs with different lignin contents. (**A**) Dispersion in DMAc solution: (a) CNFs, (b) 5-LCNFs, (c) 9-LCNFs, and (d) 14-LCNFs; (**B**) TEM morphology: (a) CNFs, (b) 5-LCNFs, (c) 9-LCNFs, and (d) 14-LCNFs; (**C**) Thermal stability; (**D**) Contact angles for three different probing liquids.

To investigate the stability of the LCNFs suspension, DMAc suspensions with 0.01 g/mL CNFs, 5-LCNFs, 9-LCNFs, and 14-LCNFs were kept in plastic centrifuge tubes for one week at room temperature (Figure 1A). The LCNFs containing lignin were well-dispersed in DMAc and the color of the suspensions was darker with increasing lignin content. The CNFs without lignin showed stratification through the flocculation process. This is because the high hydrophilicity of the CNFs without lignin means that they easily form agglomerates in DMAc. Furthermore, increasing the hydrophobicity enhances the dispersion stability of the LCNFs. The morphologies of the dispersed LCNFs suspensions with different lignin contents were evaluated by TEM analysis (Figure 1B). CNFs and LCNFs with widths of 50 nm and lengths of several hundred nanometers are clearly observed in the images. The research of Rojo showed the AFM images of the lignin containing fibrils appeared to be quite similar than CNF. However, some small, globular shaped particles could be identified in addition to the fibrils in the lignin containing fibrils sample. These particles are predominantly located between the cellulosic nano-fibers, forming complex composite structures with the 30 fibrils [26]. These observations are consistent with the role of lignin in native wood cell wall, where it exists as a stiff phase between cellulosic fibers. Thus, in our work, the LNCFs with different lignin contents show the same morphology by TEM, while the globular shaped particles (lignin) could not be obviously observed. The high lignin could form complex composite structures in LCNFs. Thus, the LCNFs are more likely to form a cross-linked network structure with increasing lignin content. In addition, The thermal stability of the LCNFs was characterized by TGA, and the thermal degradation curves of the CNFs, 5-LCNFs, 9-LCNFs, and 14-LCNFs are shown in Figure 1C. The results reveal that the thermal stability is highly affected by the lignin content. The thermal degradation onset temperature (*T*_onset_) of the LCNFs decreases with increasing lignin content (14-LCNFs < 9-LCNFs < 5-LCNFs < CNFs) and the residue weight of the LCNFs also increases. This can be attributed to the high thermal stability of lignin [27,28]. However, the weight loss of lignin is greater than that of cellulose under low temperature (*T* < 300°C) [29]. When the temperature is above 400 °C, cellulose is almost completely degraded, whereas lignin shows low weight loss. The presence of aromatic char, originating from the lignin, is responsible for the beneficial effect on the thermal stability of the CNFs. The average CAs for three probing liquids were measured on LCNFs with different lignin contents, and these were used in surface energy evaluation, as shown in Figure 1D, and summarized in Table 1. The highest CAs were obtained with water, whereas ethylene glycol gave the lowest CA in most of the cases. For a given liquid, the CA increases with increasing lignin content. Acid–base theory can be used to evaluate the surface energy components using the CA value. Using the acid–base framework to describe the surface energy has been found to be suitable to explain the properties of the wood surface, and it gives detailed information about the surface chemistry [26,30]. As a characteristic parameter, the surface free energy has a large effect on many interfacial processes, such as absorption, wetting, and adhesion. The literature surface free energy parameters for the test liquids and the surface energy components of the LCNFs, calculated by the acid–base approach, are listed in Table 1. The total surface free energy of the LCNFs with different lignin contents ranges from 47.6 to 53.6 mJ/m^2^, which is consistent with the range of 43.1–53.7 mJ/m^2^ previously reported by Peng et al. [31]. The total surface energy decreases with increasing lignin content, from 51.7 mJ/m^2^ for the 5-LCNFs sample, to 46.7 mJ/m^2^ for the 14-LCNFs sample. Therefore, lignin decreases the surface energy of the nano-fibrils, which is expected because of the large percentage of C–C and C–H bonds, and the lower ratio compared with cellulose [32]. The increasing hydrophobicity of the LCNFs, with increasing lignin content, contributes to the compatibility with the hydrophobic polymer matrix [33,34].

### 3.2. PLA/LCNFs Composite Films

#### 3.2.1. FTIR Spectroscopy

**Figure 2:**
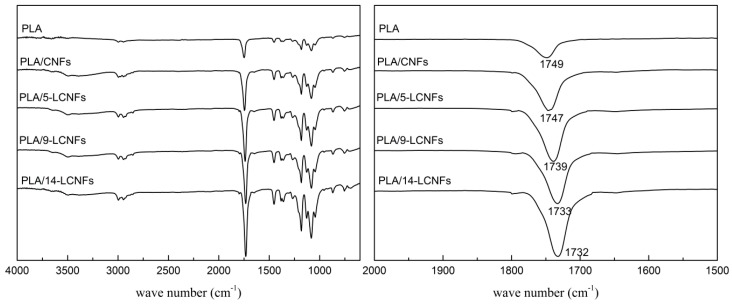
FTIR spectra of PLA, the PLA/CNFs composite, and PLA/LCNFs composites with different lignin contents.

The FTIR spectra of PLA and the PLA/CNFs and PLA/LCNFs composites are shown in Figure 2. The PLA/CNFs composite has the same peaks as pure PLA except for a stronger and wider peak around 3343–3432 cm^−1^, which can be attributed to the –OH groups of the pyranose rings of the CNFs [35]. This is because of the physical blending mode of PLA and CNFs. Furthermore, the C=O stretching vibration peak at 1749 cm^−1^ for PLA shifts in the low wave number direction by two cm^−1^ for the PLA/CNFs composite. This can be ascribed to the weak interaction between PLA and CNFs, which is not sufficient to form strong interface interactions. Comparing the spectra of the PLA/5-LCNFs, PLA/9-LCNFs, and PLA/14-LCNFs composites, the intensity of the –OH stretching vibration peak shows decreases with increasing lignin content. The C=O stretching vibration peak at 1749 cm^−1^ for PLA shifts in the low wave number direction by 10 cm^−1^ for the PLA/5-LCNFs composite (1739 cm^−1^), 16 cm^−1^ for the PLA/9-LCNFs composite (1733 cm^−1^), and 17 cm^−1^ for the PLA/14-LCNFs composite (1732 cm^−1^). The intensity of the C=O peak also increases with increasing lignin content. This suggests that there is a strong interaction between the LCNFs and PLA, which indicates that lignin improves the compatibility between the CNFs and PLA. Furthermore, the interaction increases with increasing lignin content.

#### 3.2.2. Mechanical Properties

**Figure 3:**
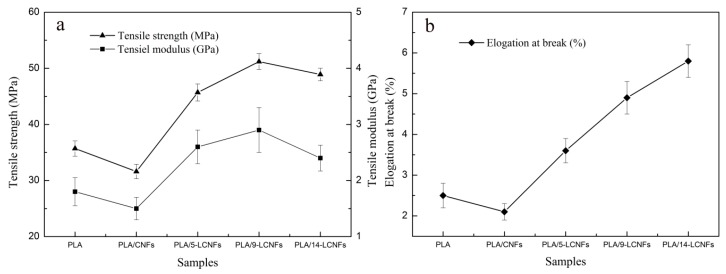
Mechanical properties of PLA and the PLA/LCNFs composites determined by tensile testing. (**a**) Tensile strength and modulus and (**b**) elongation at break.

Tensile strength evaluation is important to investigate the fracture flexibility of polymer composites. As shown in Figure 3a, the tensile strength and modulus of the PLA/LCNFs composite tend to increase with increasing lignin content for low lignin content (0 to 9 wt %), but they slightly decrease for high lignin content (14 wt %). The tensile strength and modulus decrease from 35.7 MPa and 1.8 GPa, for PLA, to 31.6 MPa and 1.5 GPa for the PLA/CNFs composite, respectively. For the PLA/LCNFs composites, there are statistically significant changes in the tensile strength and modulus for lignin contents of five and nine wt %, compared with the PLA/CNFs composite. Compared with pure PLA, the PLA/5-LCNFs and PLA/9-LCNFs composites show statistically significant improvements in the tensile strength of 28% (45.7 MPa) and 37% (48.9 MPa), and tensile modulus of 44.4% (2.6 GPa) and 61.1% (2.9 GPa). These observations can be attributed to the better compatibility of the LCNFs than the CNFs with PLA. Moreover, the strong hydrogen-bonding interaction between lignin and cellulose, as well as the van der Waals forces and polar interaction of lignin with PLA, makes it possible for good adhesion between the LCNFs and PLA. However, the tensile strength and modulus of the PLA/14-LCNFs composite (5.5% and 17.2%, respectively) is slightly lower than those of the PLA/9-LCNFs composite. This is probably because, even though the interface interaction between the LCNFs and PLA improves with increasing lignin content in the LCNFs, the mechanical properties of the LCNFs are also affected by the lignin content.

The elongation at break values of the PLA/LCNFs composites are shown in Figure 3b. The results suggest that the flexibility of PLA increases by blending with LCNFs. The increasing flexibility of the PLA/LCNFs composites with increasing lignin content results in an increase in the elongation at break percentage.

#### 3.2.3. Morphological Analysis of Fracture Surface

The effect of the lignin content on the miscibility between the LCNFs and PLA can be investigated by morphology analysis. The solubility parameter can be used to estimate the miscibility of polymer blends. The solubility’s of PLA and lignin are 20.2 and 19.02 MPa^1/2^, respectively [36], which indicates that there should be good miscibility between PLA and lignin. When LCNFs are blended with PLA, lignin can act as a compatibilizer to form a uniform blend system.

**Figure 4:**
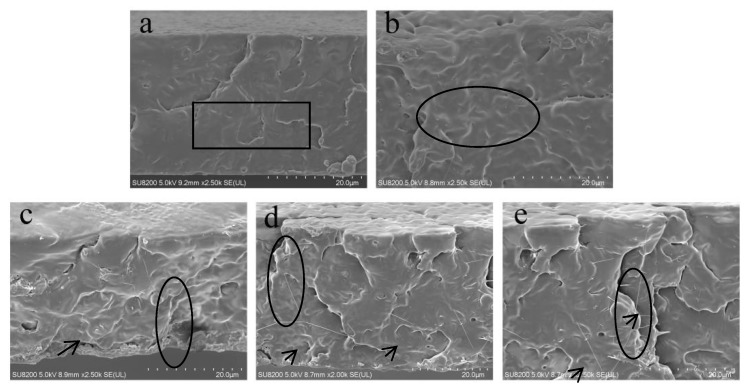
SEM images of the fractured surfaces after tensile testing: (**a**) PLA and the (**b**) PLA/CNFs, (**c**) PLA/5-LCNFs, (**d**) PLA/9-LCNFs, and (**e**) PLA/14-LCNFs composites.

To better understand the results obtained by the tensile tests, the fractured samples were observed by SEM. Figure 4a–e show the representative SEM images of the cross-sectional regions of PLA and the PLA/CNF, PLA/5-LCNFs, PLA/9-LCNFs, and PLA/14-LCNFs composites, respectively. PLA has relatively smooth fractures, which indicates that PLA is prone to brittle fracture [37]. After CNFs are added to PLA, the fracture surface of the PLA/CNF composite becomes irregular because of plastic deformation. The brittle rupture of PLA changes to ductile rupture with addition of CNFs, whereas the poor dispersion state of CNFs in the PLA matrix results in interfacial compatibility and decreases the mechanical properties of the PLA/CNF composite, compared with pure PLA. When LCNFs with different lignin contents are added to the PLA matrix, the fracture surfaces of the PLA/LCNFs composites (Figure 4c–e) show a different microstructure: At lower lignin content (5 wt%), samples showed roughness and litter wire drawing phenomenon in Figure 4c, while increasing the lignin content (14 wt%), the samples showed obvious tough character and the wire drawing phenomenon [38]. The results indicate that increasing the lignin content of the LCNFs promotes ductile rupture of PLA. From the SEM results, we can conclude that lignin and the lignin content plays important roles in improving the performance of PLA.

#### 3.2.4. Thermal Properties

**Figure 5:**
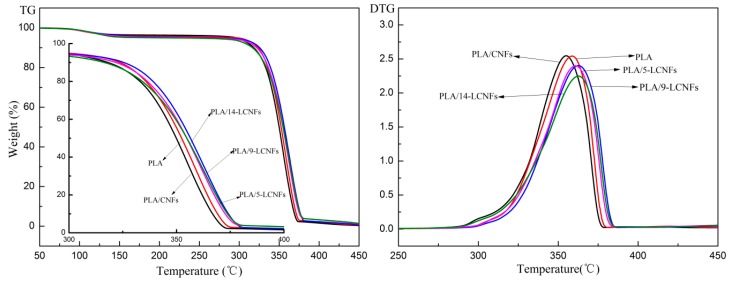
TG and DTG curves of PLA and the PLA/LCNFs composites.

In general, the thermal stability of polymers can be improved by blending with nano-fillers. The dispersion state of the nano-filler in the polymer matrix and the interaction between them significantly affect the thermal stability. The TG and derivative TG (DTG) curves of PLA and the PLA/LCNFs composites are shown in Figure 5, and the degradation parameters are summarized in Table 2. The onset temperature (10% weight loss, *T*_10%_) of thermal degradation decreases after blending with CNFs, while it increases by blending with LCNFs. This can be attributed to formation of a cross-linked structure, which reduces the chain mobility and inhibits chain unzipping during propagation of the degradation process [39]. As shown in Figure 5, the lignin content has a clear effect on the *T*_max_ (The temperature of the maximum weight loss) value of the PLA/LCNFs composites. The *T*_max_ value of the PLA/9-LCNFs composite is 5 °C higher than that of PLA. The lignin content affects the *T*_max_ value, and the thermal stability of the PLA/LCNFs composite improves with increasing lignin content in the considered temperature region. The thermal stability of the LCNFs decreases with increasing lignin content, so the PLA/14-LCNFs composite has lower thermal stability than the PLA/9-LCNFs composite. It can be concluded that lignin has a positive effect on the miscibility between PLA and the LCNFs, and increasing the lignin content also affects the thermal resistance of the LCNFs. In addition, the residue mass of the PLA/LCNFs composite in the thermal degradation process increases with increasing lignin content.

**Figure 6:**
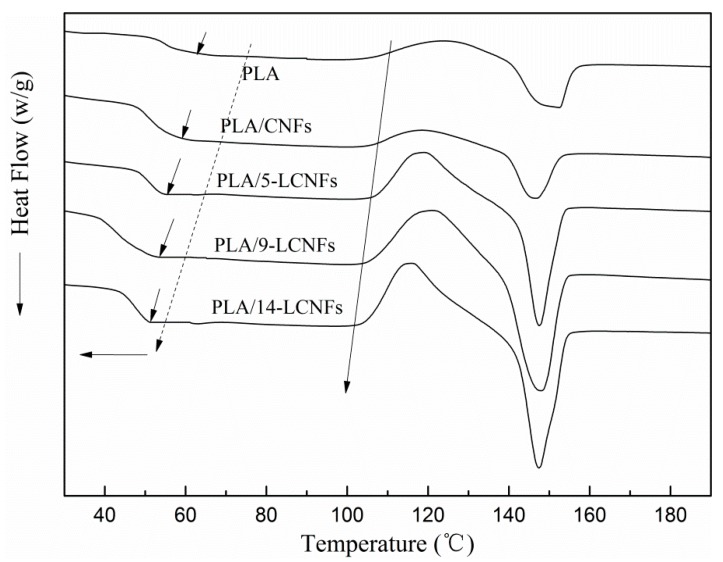
DSC curves of PLA and the PLA/LCNFs composites.

The glass transition is a complex phenomenon that depends on a number of factors, such as the chain flexibility, the molecular weight, branching, cross-linking, intermolecular interactions, and steric effects [40]. The thermal behavior of PLA and the PLA/CNFs, PLA/5-LCNFs, PLA/9-LCNFs, and PLA/14-LCNFs composites were determined by DSC measurements (Figure 6). The thermal properties of these materials are given in Table 3. The glass transition temperature (*T*_g_) of pure PLA is 61.2 °C, and the PLA/CNFs composite has a slightly lower *T*_g_ of 60.8 °C. This can be ascribed to the fact that the CNFs and PLA do not form strong interfacial interactions. However, the PLA/LCNFs composites have considerably lower *T*_g_ values than PLA. The *T*_g_ value of the PLA/14-LCNFs composite is 52.6 °C. This probably indicates that a relatively small amount of LCNFs is sufficient to change the PLA chain mobility within the glass transition region. This observation shows that there are strong interfacial forces between the LCNFs and PLA, and the lignin content has a positive effect on compatibility.

Blending with CNFs and LCNFs decreases the crystallization temperature (*T*_c_) of PLA, and *T*_c_ decreases with increasing lignin content, as shown in Table 3. The lower *T*_c_ in heat flow indicates that the crystallization rate of PLA markedly increases. The results show that the CNFs in the LCNFs can be used as a nucleating agent in PLA composites.

The melting temperature (*T*_m_) of all of the PLA composites is about 148 °C (Table 3). This indicates that the lignin content plays an important role in increasing the melting enthalpy (*ΔH*_m_) of the LCNFs-reinforced PLA matrix (Table 3). The degree of crystallinity increase from 12.1% for pure PLA to 14.7% for the PLA/CNFs composite, 17.9% for the PLA/5-LCNFs composite, 18.7% for the PLA/9-LCNFs composite, and 17.5% for the PLA/14-LCNFs composite. There is a significant increase in the degree of crystallinity of PLA when high lignin content LCNFs are used, which is because the nucleating agent concentration in the LCNFs is low for high lignin content (14-LCNFs).

#### 3.2.5. Dynamic Mechanical Properties

**Figure 7:**
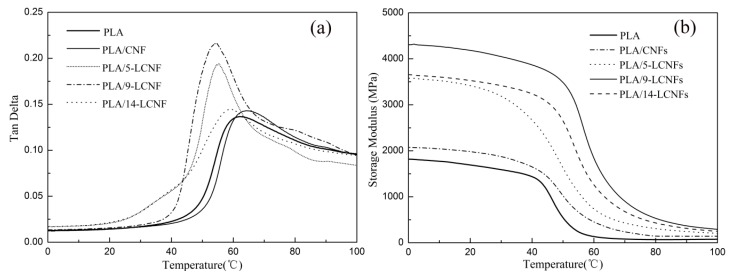
(**a**) Storage modulus and (**b**) tan Δ versus temperature for PLA and the PLA/LCNFs composites.

DMA is a useful technique to investigate the relationship between the structure and viscoelastic behavior of polymers and polymer-nano-filler composites. The storage modulus (*E*′) indicates the tendency and ability of energy storage in materials, and it is also directly related to the Young’s modulus. Figure 7a shows the plots of *E*′ versus temperature for pure PLA and the PLA/LCNFs composites with various lignin contents. When LCNFs are added, *E*′ is significantly higher in the whole temperature range studied. For example, *E*′ at 20 °C increases from 1800 MPa for pure PLA to 3650 MPa for the PLA/9-LCNFs composite. The increase in *E*′ can be explained by the reinforcing effect provided by lignin of the LCNFs on the PLA matrix. The thickness of the interface is affected by the lignin content and it affects the transition of stress, so the PLA/14-LCNFs composite has a lower *E*′ than the PLA/9-LCNFs composite, which is consistent with the mechanical strength results.

The effect of LCNFs with different lignin contents on the damping behavior of PLA was investigated by plotting tan Δ against temperature (Figure 7b). The tan Δ peak shifts to lower temperature with increasing lignin content in the LCNFs. The tan Δ peaks for the PLA/CNFs composite, PLA, and the PLA/5-LCNFs, PLA/9-LCNFs, and PLA/14-LCNFs composites are at 61, 60, 57, 55, and 54 °C, respectively. Moreover, the intensities of the tan Δ peaks for the PLA/LCNFs composites are considerably higher than the peak for pure PLA. This can be attributed to the liberation effect of the LCNFs enhancing the chain mobility of the amorphous region for the PLA/LCNFs composites with higher lignin content.

#### 3.2.6. Mechanism Analysis of the Interfacial Interaction

**Figure 8:**
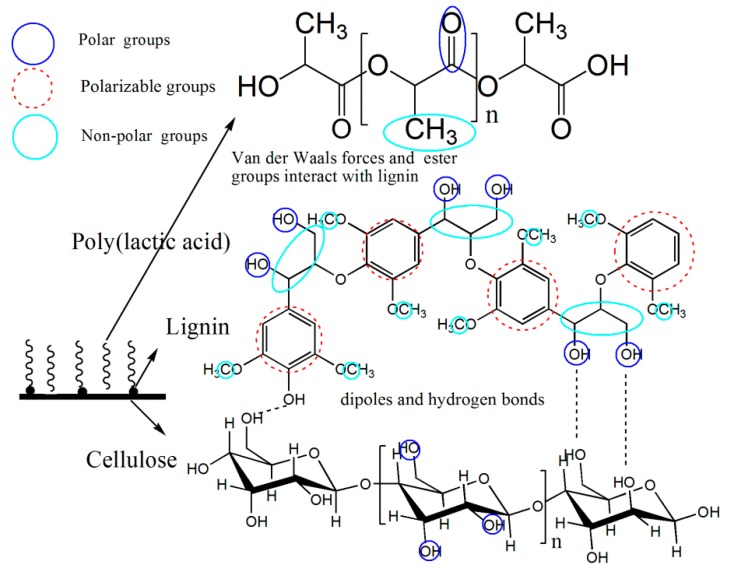
Reaction mechanism of PLA and LCNFs.

The mechanism of the interfacial interaction between the LCNFs and PLA is shown in Figure 8. Lignin can be used as an additive to improve the compatibility between CNFs and PLA. Considering the LCNFs as an ensemble, lignin combines well with cellulose through hydrogen bonds and dipole–dipole interactions, and van der Waals interactions occur among the non-polar groups of lignin and PLA. Moreover, the polar and polarizable groups of lignin, such as hydroxyl and phenyl groups, form hydrogen bonds or dispersion interactions with the ester groups of the PLA matrix, which makes it possible for lignin to be in a well-dispersed state in the PLA matrix. As a result, the interfacial compatibility between LCNFs and PLA is enhanced.

**Figure 9:**
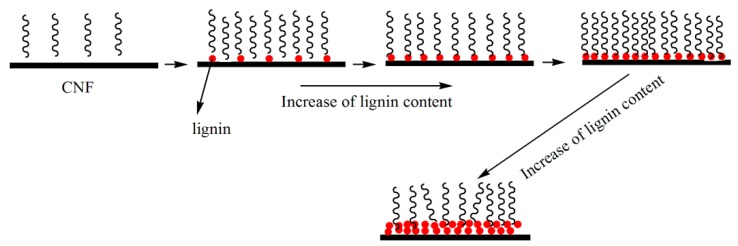
Effect of the lignin content on the compatibility between PLA and the LCNFs.

Figure 9 shows the mechanism of the effect of the lignin content on the compatibility between PLA and the LCNFs. It can be concluded that increasing the lignin content has a positive effect on the compatibility, and the interfacial layer thickness increases with increasing lignin content, which results in increased flexibility of the PLA/LCNFs composite.

## 4. Conclusions

Addition of LCNFs with different lignin contents to PLA changes the mechanical properties, thermo-mechanical properties, thermal behavior, and morphology. The tensile strength and modulus are the highest (48.9 MPa and 2.9 GPa) when the lignin content is 9 wt %. The elongation at break increases with increasing lignin content, and the thermo-mechanical properties show the same trend as the tensile strength. FTIR spectroscopy shows that there is a clear interaction between the LCNFs and the carbonyl groups of PLA. The *T*_g_ decreases from 61.2 °C for pure PLA, to 52.6 °C for the PLA/14-LCNFs composite, indicating increased flexibility with LCNFs addition with increasing lignin content. The crystallization temperature of the PLA/LCNFs composite decreases with increasing lignin content in the LCNFs, indicating that the crystallization efficiency and degree of crystallinity improve. The thermal stabilities of the PLA/LCNFs composites are higher than that of PLA, which was below 380 °C, and the thermal stability increases with increasing lignin content in the LCNFs. The PLA/9-LCNFs composite has the highest *T*_max_ value (4.4 °C higher than that of pure PLA). The cross-sectional morphologies of PLA and the PLA/LFCNFs composites show the strengthening and toughening effect of LCNFs, and the lignin content has a considerable effect on the flexibility of the PLA/LCNFs composite.

## Figures and Tables

**Table 1:** Surface free energies of LCNFs with different lignin contents.

**Test Liquids**	$\gamma_{L}$	$\gamma_{L}^{p}$	$\gamma_{L}^{d}$
Water	72.8	51.0	21.8
Formamide	58.2	18.7	39.5
Ethylene glycol	48.3	19	29.3
Solid Samples	$\gamma_{S}$	$\gamma_{S}^{p}$	$\gamma_{S}^{d}$
CNFs	53.6	4	49.6
5-LCNFs	51.7	8.5	43.2
9-LCNFs	50.5	7.8	42.7
14-LCNFs	47.6	3.5	44.1

**Table 2:** Thermal degradation data of PLA/LCNFs composites with different lignin contents.

Sample	*T*_10%_ (°C)	*T*_50%_ (°C)	*T*_max_ (°C)
PLA	318.1	352.4	358.7
PLA/CNFs	316.3	349.9	354.9
PLA/5-LCNFs	321.8	354.8	360.8
PLA/9-LCNFs	325.4	356.4	363.1
PLA/14-LCNFs	323.7	354.8	362.6

**Table 3:** DSC data of PLA and PLA/LCNFs composites with different lignin contents.

Samples	*T*_g_ (°C)	*T*_c_ (°C)	*T*_m_ (°C)	*ΔH*_m_ (J/g)	*X*_c_ (%)
PLA	61.2	124	150.1	11.7	12.1
PLA/CNFs	60.8	119	147.6	13.8	14.7
PLA/5-LCNFs	55.3	118	148.2	16.8	17.9
PLA/9-LCNFs	54.2	120	148.5	17.5	18.7
PLA/14-LCNFs	52.6	115	148.9	16.4	17.5

## Supplementary Materials

The supplementary materials are available online at http://www.mdpi.com/2073-4360/10/9/1013/s1.
