# Supplementary material for: Influence of the Lignin Content on the Properties of Poly(Lactic Acid)/lignin-Containing Cellulose Nanofibrils Composite Films
Source: Polymers (Basel). 2018 Sep 11;10(9):1013. doi: 10.3390/polym10091013 (PMC6403545; doi:10.3390/polym10091013)
Supplement: Supplementary file 1 [file polymers-10-01013-s001.pdf]

Supplementary Information

Influence of the lignin content on the properties of poly(lactic acid)/lignin-containing cellulose nanofibrils composite films

Xuan Wang [1*], Yuan Jia [1], Zhen Liu[1] and Jiaojiao Miao [2]

[1] College of Chemical Engineering, Xi'an University, Xi'an 710065, PR China; jiayu_an_happy@126.com (Y.J); liuzhen.lz @163.com (Z.L)
[2] School of Life Sciences, Northwestern Polytechnical University, Xi'an 710072, PR China; mjj.smile@163.com
* Correspondence: wangxuan201441@163.com; Tel.: +86 17791696569

Surface energy calculation

The theory of the contact angle of pure liquids on a solid responds to the widely applied Young's equation (Eq. S1):

$$\gamma_L \cos\theta = \gamma_S - \gamma_{SL} \quad \textbf{(S1)}$$

where γ_L is the experimentally determined surface energy of the liquid, θ is the contact angle, γ_S is the surface energy of the solid and γ_{SL} is the solid/liquid interfacial energy.

Surface energy values were calculated according to the acid-base theory, which also allowed to obtain the disperse, acid and base components. Acid-base theory introduces a combining rule that factors molecular consideration and theories of intermolecular forces and eliminates γ_{SL} from Young's equation. The total surface energy of a solid (γ_S) is calculated from the sum of one disperse component, associated with polar component (γ_S^P) and dispersion component(γ_S^d) (Eq. S2).

$$\gamma_S = \gamma_S^p + \gamma_S^d \quad \textbf{(S2)}$$

In addition, we derived the relations between polar component and dispersion component of liquids and solids (Eq. S3).

$$\gamma(1+\cos\theta) = 2\sqrt{\gamma_S^d \gamma_L^d} + 2\sqrt{\gamma_S^p \gamma_L^p} \quad \textbf{(S3)}$$

Where γ_L^p is the polar component of test liquids, γ_L^d is the dispersion component of test liquids. The γ_S^P and γ_S^d can be obtained by taking the γ_L^p and γ_L^d of each two test liquids in to equation S3.
